# Comparison of liver T1 estimates generated by breath-hold 3D FSPGR and free-breathing 3D radial stack-of-stars FSPGR variable flip angle T1 mapping sequences

**DOI:** 10.1016/j.ejro.2026.100790

**Published:** 2026-06-30

**Authors:** Haiyun Xu, Zhijun Hou, Ming Zhang, Ran Guo, Jialu Zhang, Guifeng Fu, Qingqing Wen, Zehua Li, Feng Tao, Shuohui Yang

**Affiliations:** a Department of Radiology, Shanghai Municipal Hospital of Traditional Chinese Medicine, Shanghai University of Traditional Chinese Medicine, Shanghai, SH, China; b Department of Hepatopathy, Shanghai Municipal Hospital of Traditional Chinese Medicine, Shanghai University of Traditional Chinese Medicine, Shanghai, SH, China; c GE Healthcare, MR Research, Beijing, PK, China; d Department of Endocrinology, Shanghai Municipal Hospital of Traditional Chinese Medicine, Shanghai University of Traditional Chinese Medicine, Shanghai, SH, China

**Keywords:** Liver, MRI, T1 mapping, Obesity

## Abstract

**Objective:**

To evaluate the feasibility of using the free-breathing (FB) radial stack-of-stars fast spoiled gradient recalled-echo (FSPGR) sequence as an alternative to the conventional breath-hold (BH) FSPGR sequence for hepatic T1 mapping across the clinical range of body mass index (BMI).

**Methods:**

From 07/2024 to 10/2024, 75 participants who underwent both FB and BH FSPGR were analyzed. Participants were stratified into 3 BMI groups. BH IDEAL IQ was also collected to enable PDFF to be evaluated. Quantitative assessments involved whole-liver T1 estimates, volumes, PDFF and the ratio of |T1_liver_-T1_sat_|/T1_liver_. A five-point Likert scale was used for qualitative assessments.

**Results:**

The ratios of |T1_liver_-T1_sat_|/T1 _liver_ in FB T1 mapping were higher than those of BH T1 mapping in the overall population and obese group (*p* both < 0.05). No significant difference was found in liver T1 estimates in patients with BH failure between sequences (*p*＞0.05). FB T1 mapping outperformed BH in artifact scores for the obese group (*p* = 0.015). Of the 3 groups, the obese group exhibited the greatest amount of artifact in BH FSPGR T1 mapping (*p* < 0.05). The FB T1 mapping demonstrated superior performance in minimizing artifacts in the lower liver region in persons with BH failure [4 (4, 5) vs. 3 (3, 4), *p* < 0.001].

**Conclusion:**

For overweight and obese patients with difficulty holding their breath during abdominal scanning, VFA FB radial stack-of-stars FSPGR T1 mapping served as a complementary and alternative method to BH sequence.

## Highlights

• FB radial FSPGR enables feasible T1 mapping for obese patients.
• Liver T1 values and volumes showed no difference between FB and BF sequences in all participants.
• FB improved image quality consistency in obese patients by reducing motion artifacts.

## 1 Introduction

Obesity is emerging as a major global health concern due to its increasing prevalence [1]. The rate of adult obesity has almost tripled worldwide since 1975, and the prevalence of obesity and the average body-mass index (BMI) have also been continuously rising with an approximately 5% increase in China due to the booming economy [2]. Patients with obesity or high BMI lead an increasing likelihood of liver diseases, particularly metabolic dysfunction-associated fatty liver disease [3]. Magnetic resonance imaging (MRI)-based T1 mapping, an advanced non-invasive method to evaluate the overall liver health and composition of liver tissue, has been proven to differentiate the degree of hepatic steatosis, liver inflammation and fibrosis [4], [5], [6].

In abdominal MRI, breath-hold (BH) T1-weighted gradient-echo sequences are standard for fast acquisition but highly susceptible to motion artifacts, which degrade image quality, obscure anatomy and lesions, and impair quantitative accuracy—especially in patients unable to sustain breath-holding [7]. The three-dimensional (3D) radial stack-of-stars k-space trajectory, combined with the Variable Flip Angle (VFA) method, has been introduced as a motion-robust alternative, enabling free-breathing (FB) abdominal imaging [8], [9], [10], [11]. This sequence samples k-space radially in the kx–ky plane and sequentially along the kz direction, providing inherent motion resilience and facilitating data acquisition without breath-holding [12], [13], [14], [15]. The proposed method is more robust to motion artifacts than Cartesian trajectories due to radial sampling in the kx-ky plane. Furthermore, incoherent aliasing artifacts created by radial sampling are well suited for compressed sensing reconstruction and retrospective motion compensation. While both BH and FB FSPGR sequences are clinically available, a direct quantitative comparison of hepatic T1 mapping between these two techniques in obese individuals—who often have limited breath-hold capacity and higher motion susceptibility—remains unexplored.

The present study was therefore designed as a technical feasibility and robustness investigation to systematically compare hepatic T1 estimates derived from BH VFA T1 mapping and FB radial stack-of-stars VFA T1 mapping in obese patients. The primary aim was to assess the quantitative agreement between the two sequences and validate the reliability of the FB approach in this challenging cohort with increased motion vulnerability. Secondary objectives included subjective evaluation of image quality and preliminary exploration of relationships between T1 values, liver volume, and proton density fat fraction (PDFF), to confirm the technical feasibility of multi-parametric hepatic characterization from free-breathing acquisition.

## 2 Materials and methods

### 2.1 Study population

This prospective study was approved by the local institutional ethics committee. Written informed consent was obtained from all participants.

**Fig. 1:**
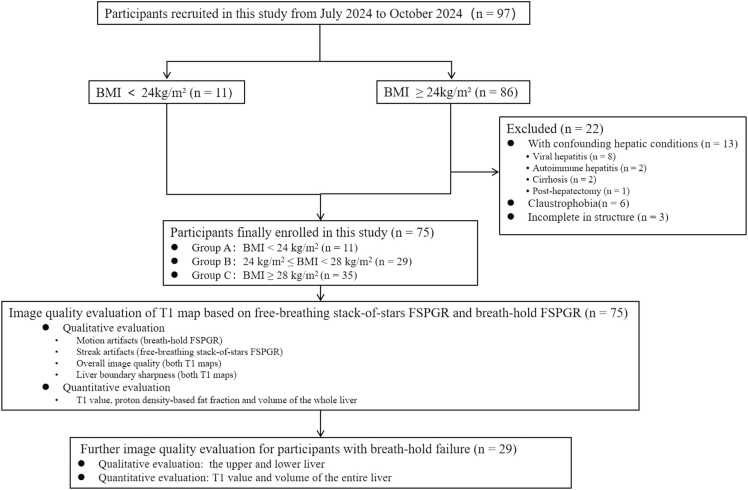
Flowchart of participants enrolled process and image quality evaluation. BMI, body-mass index; FSPGR, fast spoiled gradient recalled-echo.

A total of 97 participants were recruited between July 2024 and October 2024 in this exploratory, prospective study. Among them, 11 participants with normal BMI (< 24 kg/m^2^) were enrolled in group A as the control group. Other participants’ (n = 86) inclusion criteria were as follows: (1) visited the Department of Endocrinology and Department of Hepatopathy, diagnosed as overweight or obese; (2) BMI ≥ 24 kg/m^2^; and (3) age from 18 to 70 years old. The patients were divided into group B (overweight: 24 kg/m^2^ ≤ BMI < 28 kg/m^2^) and group C (obese: BMI ≥ 28 kg/m^2^) according to the standard grouping used in studies based on the Chinese community population [16], [17]. Exclusion criteria included patients with evidence of other liver diseases [18] (e.g., viral hepatitis, autoimmune liver diseases, drug-induced liver injury, cirrhosis, malignant liver tumor, liver post-hepatectomy, and decompensated liver diseases), those with MRI contraindications and those with incomplete coverage of the entire liver on MRI scans. Through a comprehensive medical history review, 13 cases with confounding hepatic conditions that could affect hepatic T1 estimates were excluded, including 8 with viral hepatitis, 2 with autoimmune liver diseases, 2 with cirrhosis, and 1 post-hepatectomy patient, to ensure that observed differences in T1 estimates and image quality were due to technical variations rather than underlying pathologies. Additionally, 6 participants were excluded for claustrophobia, 3 for incomplete liver coverage, resulting in 64 analyzable cases of overweight and obese. Finally, a total of 75 participants were enrolled in the study and divided into three groups (Group A: 11, Group B: 29, and Group C: 35) (Fig. 1).

### 2.2 MRI acquisition

All examinations were conducted on a 3.0-T MR scanner (Signa Premier, GE Healthcare, Milwaukee, WI, USA) with 70 cm diameter large bore friendly accommodating obese patients. A flexible 30-channel coil (AIR™ Coil, GE Healthcare) and 60-channel embedded table coils were used for signal reception and transmit. Participants were asked to lie in the supine position oriented with the head-foot direction on the scanning table. The field of view selected should be big enough for optimum anatomic depiction of the entire liver and diaphragm. To standardize breath-hold position across the four flip angle acquisitions, we implemented the following protocol: (a) Breath-hold Training: Before scanning, all patients underwent consistent coaching to practice the breath-hold maneuver. (b) Abdominal Compression: A commercially available elastic compression belt was firmly secured around the upper abdomen to reduce respiratory amplitude and mechanically restrict diaphragm movement, thereby reducing corresponding motion artifacts. (c) Standardized Instruction: Patients were instructed to hold their breath at the end of a normal expiration, aiming for a consistent and reproducible diaphragmatic position for each acquisition. They were also instructed to maintain steady breathing during the VFA FB sequences. Each flip angle acquisition for the VFA BH 3D FSPGR and FB 3D FSPGR scan was performed as a separate BH and FB. To ensure the participant's stable breathing during the previous scan, the FB stack-of-stars FSPGR sequence was scanned before the BH FSPGR sequence. Abdominal breathing was monitored via bellows for all patients. If during a set of breath-holding scans, the participant's abdominal breathing waveform was observed to convert from a relatively flat line to a wavy pattern, and obvious artifacts were observed in the source T1 FSPGR images, it was determined that the breath hold was not maintained throughout the scan.

T1 mapping calculated from FB sequence and BH sequence was obtained by four identical sets of 3D FSPGR with variable flip angles (3°, 6°, 9°, and 12°) and same fixed parameters, which minimized the differences in scanning protocol and ensured the comparison disparity only caused by sequences themselves and distinct breath-control method. The fixed parameters were shown in Table 1. Crucially, as the actual flip angle achieved in tissue is determined by the local radiofrequency transmit field (B1 +), spatial inhomogeneities in this B1 + field can introduce significant errors into T1 quantification [19]. Therefore, B1 correction was applied to ensure the accuracy of T1 mappings. GE provided B1 mapping sequence protocol. The 2D B1 mapping was acquired in the same area (voxel size of 3 × 3 × 8 mm^3^) with BH for correction use.

**Table 1:** MRI sequence parameters for liver T1 mapping.

	3D FB FSPGR		3D BH FSPGR
TR/TE (ms)	4.6/2.1		
FOV (mm)	400 × 400		
Slice thickness (mm)	4.0		
Acquisition Matrix	260 × 260		
Voxel size (mm)	1.5 × 1.5 × 4 mm³		
Flip angle	3º, 6º, 9º, and 12º		
Bandwidth (Hz/pixel)	41.67		
Number of excitations	2	1	
Scan Time per Flip Angle Acquisition	1 min 28 s to 2 min 3 s	16 s to 22 s	

MRI, Magnetic Resonance Imaging**;** 3D, three-dimensional; FB, free-breathing; FSPGR, fast spoiled gradient recalled-echo; BH, breath-hold; TR, repetition Time; TE, echo time; FOV, field of view.

Furthermore, the conventional sequences of abdominal MR examination, including axial T1-weighted imaging, T2-weighted imaging, and iterative decomposition of water and fat with echo asymmetry and least-squares estimation (IDEAL-IQ) sequence, were performed for all groups.

### 2.3 MRI post-processing

Two sets of VFA T1W images acquired from FB FSPGR and BH FSPGR, accompanied by B1-mapping, were transferred off-line to proceed post-processing and calculate T1 mapping within MATLAB (MathWorks, Natick, MA). All datasets, including B1-mappings and VFA T1-weighted images, were co-registered and re-sliced using a rigid-body transformation implemented in SPM12 toolbox (Functional Imaging Laboratory, London, UK). The B1-mapping was aligned to the corresponding T1-weighted dataset and smoothed by a 12 mm isotropic smoothing kernel as preparation for T1 mapping calculation. The smoothing on B1-mappings and the robustness of radial stack-of-stars acquisitions to motion helped minimize residual misregistration effects. T1 value estimates were then calculated as literatures presented [20], [21] where the SI could be derived from equilibrium magnetization (*M*_0_), longitudinal relaxation (T1), TR and FA (*α*) in the following equation:

(1) $$\mathrm{SI}=M_{0}\cdot \frac{\sin\alpha \cdot \left(1-E_{1}\right)\cdot E_{2}}{1-E_{1}\cdot \cos\alpha }$$

In this case, *E*_1_ = exp (-TR/T1) and *E*_2_ = exp (-TE/T2*). Considering TE ≪ T2*, the Eq. (1) can be written in a linear form:

(2) $$\frac{\mathrm{SI}}{\sin\alpha }=E_{1}\cdot \frac{\mathrm{SI}}{\tan\alpha }+M_{0}\cdot \left(1-E_{1}\right)$$

To solve Eq. (2), TR is constant during the acquisitions of different FAs, leading to a series of equations could be easily solved in a linear least square fit [22], [23].

**Fig. 2:**
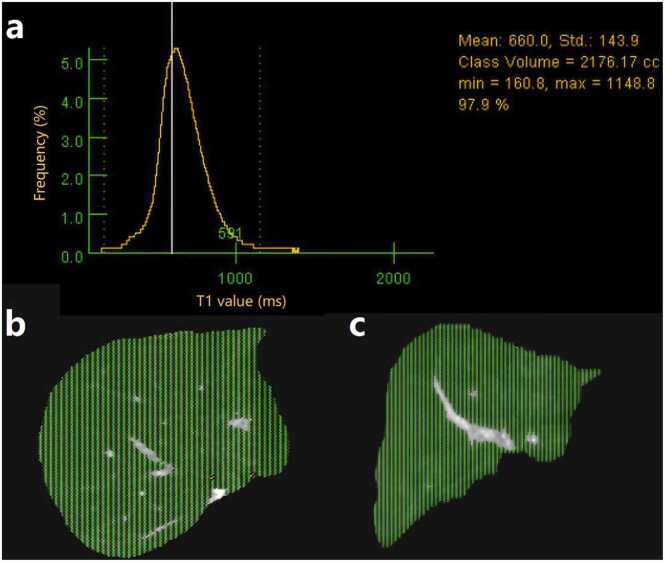
Illustration of entire-liver segmentation for a 35-year-old male T1 map results on the image signal processor post-processing platform. (a) Manually alterations by adjusting the threshold of T1 value. (b) Axial image of the segmented entire liver. (c) Coronal image of the segmented entire liver. The mean and standard deviation of T1 value for the entire hepatic tissue was 660.0 ± 143.9 ms. The volume of the entire hepatic tissue was 2176.17 cm^3^.

After the two sets of T1 mapping were generated, they were imported into Advantage Workstation 4.7 (GE Healthcare, Milwaukee, WI, USA) for further semi-automatic segmentations. The semi-automated tracing and segmentation of the entire liver for all groups was accomplished by delineating along the entire surface of the liver parenchyma to avoid obvious artifacts around the liver edges. Manual adjustments were necessary for obtaining clear margins and excluding the main portal vein, inferior vena cava, and the gallbladder through adjusting the threshold of T1 value. Once the hepatic tissue was segmented on the T1 mappings, the entire hepatic T1 value estimate and volume were automatically calculated. An example of entire-liver semi-automatic segmentation for a 35-year-old male was shown in Fig. 2.

**Fig. 3:**
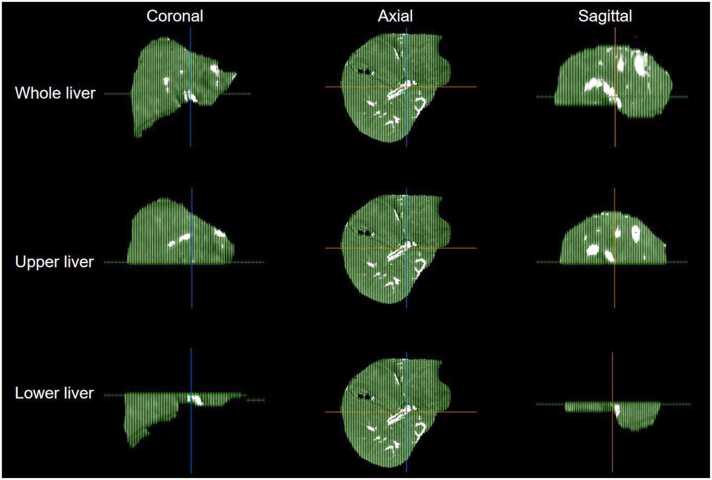
Demonstration of the segmentation of the whole liver, upper liver and lower liver.

For patients who are unable to hold their breath throughout the sequence, further segmentation of the upper and lower livers was required. Segmentation of each portion followed the same semi-automated approach as used for the whole liver. The upper half of the liver was defined as the liver tissue from the level of the first hepatic hilum until the level of the upper pole of the liver. And the lower liver was the other half part of the liver tissue (Fig. 3). It is worth noting that the entire liver data was obtained through semi-automatic segmentation of the entire liver, rather than by summing up the data of the upper and lower parts.

**Fig. 4:**
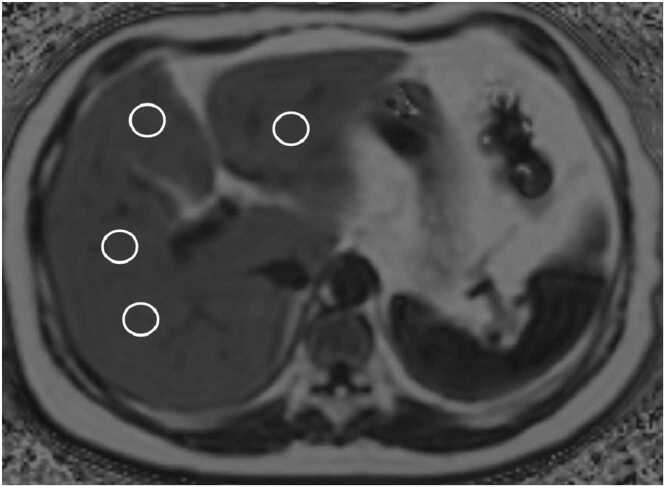
Representative proton density fat fraction (PDFF) quantification in hepatic tissue from a 55-year-old female participant.

The quantitative proton density fat fraction (PDFF) maps derived from IDEAL-IQ sequence used for the evaluation were automatically generated on the scanner console by the vendor software. Four regions of interests (ROI) measuring 200 mm^2^ each were drawn on the PDFF maps of liver (two in the right lobe and two in the left lobe) at the level of the porta hepatitis [24] (Fig. 4), and the average values were calculated for all participants. Care was taken to avoid blood vessels, artifacts, and the liver border by maintaining a minimum distance of 5 mm from these structures.

### 2.4 Quantitative and qualitative image analysis of FB Stack-of-stars FSPGR and BH FSPGR

Quantitative analyses were independently performed by two blinded radiologists (with 6 and 9 years of post-training experience in abdominal imaging, respectively) on: (1) T1 value estimates and volumes of the entire liver (for all participants), upper and lower liver (for participants with BH failure) from the two sets of T1 mappings, and (2) entire liver PDFF based on IDEAL-IQ sequence (for all participants). The average processing time per observer was approximately 3 min for whole-liver T1/volume analysis, 5 min for bi-segmental (upper/lower) liver analysis, and 2 min for whole-liver PDFF analysis. Inter-observer agreement will be assessed. The final measurements from the two readers were averaged. The correlations between the liver volume, T1 estimates, PDFF and BMI (calculated based on weight and height measured at the time of scan) were analyzed.

Contrast-to-noise ratio (CNR) is usually used to objectively quantify the quality of the image. A ratio was defined to quantify the stability of the T1 estimates, which is calculated by a formula similar to CNR: the absolute differences between the T1 estimates of the liver and abdominal subcutaneous adipose tissue (sat) to the T1 value of the liver (|T1_liver_-T1_sat_|/T1 _liver_). T1 _liver_ was obtained by automatic calculation after the segmentation. T1_sat_ were measured by an ROI with a diameter of 1 cm placed in the artifact-free abdominal subcutaneous adipose tissue, respectively. The average ratios from the two T1 mappings were compared.

**Fig. 5:**
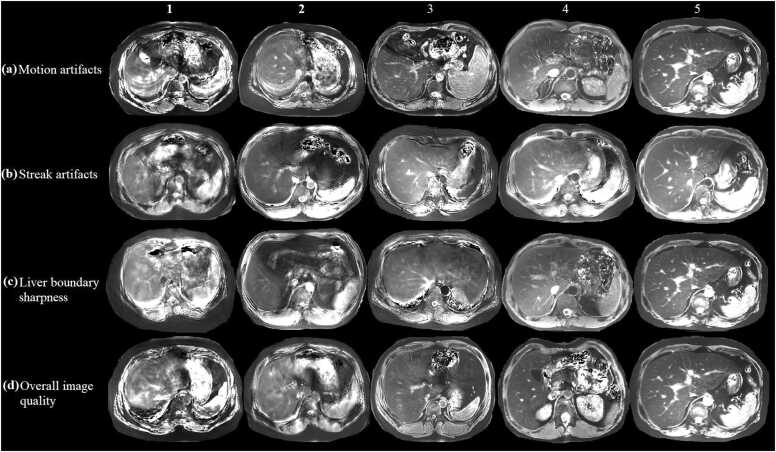
Representative graded images for qualitative visual assessment across four evaluation dimensions. For grouped subpanels: all motion-artifact samples (row a) originate from breath-hold (BH) FSPGR acquisitions; all streak-artifact samples (row b) derive from free-breathing (FB) stack-of-stars FSPGR; liver boundary sharpness (row c) and overall image quality (row d) include representative cases from both BH and FB sequences. Columns 1–5 display images graded from the poorest diagnostic quality (score = 1) to optimal image appearance (score = 5): (a) Motion artifacts (BH-only source): 1 = extensive motion artifact, unreadable images; 2 = obvious motion artifacts in both the upper and lower segments of the liver; 3 = obvious motion artifacts only in the lower liver segment; 4 = mild motion artifacts in the lower liver segment; 5 = no obvious motion artifact in the whole liver; (b) streak artifacts (FB-only source): 1 = extensive streak artifact, unreadable images; 2 = obvious streak artifacts but interpretable; 3 = moderate streak artifacts; 4 = mild streak artifacts; 5 = no obvious streak artifact in the whole liver [owing to inherent radial undersampling of FB radial sampling, complete absence of streaking (grade 5) was not observed on FB acquisitions, with grade-5 reference benchmark referenced to the artifact-free standard of motion scoring]; (c) liver boundary sharpness (mixed BH + FB cases): 1 = extensive blur, unreadable image; 2 = in addition to grade 3 performance, blur in the lower liver margin; 3 = obvious blur between the lateral segment of the liver and the stomach; 4 = mild blur between the lateral segment of the liver and the stomach; 5 = no blur; (d) overall image quality (mixed BH + FB cases), comprehensively scored combining artifact severity and hepatic border definition: 1 = severe artifacts with extensive blur; 2 = in addition to grade 3 performance, obvious artifacts with obvious blur in the lower liver margin; 3 = obvious artifacts and/or obvious blur between the lateral segment of the liver and the stomach; 4 = mild artifacts or mild blur between the lateral segment of the liver and the stomach; 5 = artifact-free images with well-demarcated liver boundaries.

The qualitative image evaluations of (1) motion artifacts (BH FSPGR), (2) streak artifacts (FB stack-of-stars FSPGR), (3) liver boundary sharpness, and (4) overall image quality, respectively, were made using a five-point Likert-type scale (Fig. 5). The motion artifact appeared as a ghost or an artifact parallel to an edge of the liver in BH FSPGR, while rarely observed in FB stack-of-stars FSPGR. In FB stack-of-stars FSPGR, artifacts were primarily prevalent in the form of streak artifacts instead. So, motion artifacts and streak artifacts were assessed in BH FSPGR and FB stack-of-stars FSPGR, respectively. The qualitative image evaluation was made for the entire liver of all participants, as well as for the upper and lower parts of the liver of participants with BH failure on the two T1 mappings by the two radiologists. Inter-observer agreement was evaluated, with final measurements derived from the mean values of both radiologists' independent assessments.

Due to inherent differences between sequence types, complete blinding of the two sets of T1 mappings was technically unfeasible. However, we implemented rigorous safeguards: (1) readers were blinded to all patient data; (2) sequences were evaluated separately in randomized order; (3) standardized grading criteria were used.

### 2.5 Statistical analysis

All data were analyzed using SPSS version 29.0 (IBM Corp., Armonk, NY, USA). The Shapiro-Wilk test was used to assess the normality of the variables. Normally distributed data were expressed as mean ± standard deviations (SD), while non-normally distributed data and hierarchical data were expressed as median (P_25_, P_75_). The weighted Kappa analysis and intraclass correlation coefficients (ICC) were used to check the 2 readers’ consistency. The Independent-samples *t*-tests were used to compare the normally distributed data of T1 estimates between the upper and lower liver. One-way analysis of variance (ANOVA) with the Least Significant Difference (LSD) post hoc test was adopted for multiple-group comparisons, and LSD was used for post-hoc multiple-comparison correction among the three groups. The paired-sample *t*-tests were used to compare the normally distributed data between FB stack-of-stars FSPGR and BH FSPGR. Categorical data were also compared by Chi-square test. Hierarchical data between two different groups or sequences were compared by the paired Wilcoxon test and the Mann-Whitney *U* test. Spearman’s correlation coefficient (skewed distribution data) and Pearson’s correlation coefficient (normal distribution data) were used in the correlation analysis between BMI, liver PDFF, T1 estimates and volumes. All statistical comparisons, including correlations, adhered to the principle of within-subject methodological consistency. The whole-liver and regional data in any single analysis were not mixed. The Spearman or Pearson correlation analysis (bilateral sides) was performed with |r| < 0.3 as a weak association, 0.3 ≤ |r| < 0.5 as a mild association, 0.5 ≤ |r| < 0.8 as a moderate association, |r| ≥ 0.8 as a marked association, and |r| > 0.95 as a highly marked association. A two-tailed *p* value < 0.05 was considered statistically significant.

## 3 Results

### 3.1 Study population

The general clinical characteristics of 75 subjects were evaluated in Table 2. In Groups A, B and C, 6, 22 and 28 participants were diagnosed with fatty liver for PDFF over 5%, respectively. During BH FSPGR scanning, 29 participants (2 in Group A [18.2%], 12 in Group B [41.4%] and 15 in Group C [42.9%]) failed to hold their breath.

**Table 2:** General clinical characteristics of the study population.

**Parameter**	**Group A**	**Group B**	**Group C**	***χ²/F/Z***	***p***
N (Male: Female)	11 (7: 4)	29 (14: 15)	35 (13: 22)	2.536	0.281*
Age (years)	41.91 ± 8.86	45.31 ± 8.85	42.43 ± 8.07	1.130	0.329^†^
Weight (kg)	60.68 ± 8.53	71.43 ± 7.64	90.63 ± 11.16	55.052	**< 0.001**^**†#**^
Height (m)	1.64 (1.57, 1.75)	1.64 (1.60, 1.71)	1.70 (1.63, 1.77)	5.268	0.072^‡^
PDFF (%)	8.218 ± 8.847	12.058 ± 8.714	14.934 ± 9.608	5.718	0.057
BMI (kg/m^2^)	22.03 ± 1.42	26.10 ± 1.20	31.34 ± 2.82	96.929	**< 0.001**^**†##**^

Data are shown as mean ± SD or Median (P_25_, P_75_).

*, Chi-square test (*χ*²); ^†^, one-way ANOVA with Least Significant Difference test (*F*); ^‡^, Kruskal-Wallis test (*Z*).

^#^, Group A < Group B < Group C (*p* all < 0.005); ^##^, Group A < Group B < Group C (*p* all < 0.005).

PDFF, proton density-based fat fraction; BMI, Body Mass Index.

### 3.2 Quantitative evaluation of the two T1 mappings

The inter-readers consistencies of all quantitative assessments were good to excellent for both sequences (ICC = 0.741–0.928, *p* < 0.001) (Table 3).

**Table 3:** Inter-readers reliability (ICC) for quantitative evaluation for FB stack-of-stars FSPGR and BH FSPGR.

**Sequence**	**Quality Parameter**	**ICC**	***95% CI***	***p***
IDEAL-IQ	PDFF	0.994	0.991–0.996	**< 0.001**
FB stack-of-stars FSPGR	T1 estimates	0.822	0.719–0.888	**< 0.001**
	volumes	0.864	0.793–0.912	**< 0.001**
	|T1_liver_-T1_sat_|/T1_liver_	0.858	0.783–0.908	**< 0.001**
BH FSPGR	T1 estimates	0.906	0.856–0.940	**< 0.001**
	volumes	0.864	0.785–0.914	**< 0.001**
	|T1_liver_-T1_sat_|/T1_liver_	0.922	0.880–0.950	**< 0.001**

FB, free-breathing; FSPGR, fast spoiled gradient recalled-echo; BH, breath-hold; CI, Confidence Interval; IDEAL-IQ, PDFF, proton density-based fat fraction; ICC, Intraclass correlation coefficients; Two readers’ experience: two radiologists with 6- and 9-years’ abdominal diagnosis experience.

T1 estimates and volumes of the entire liver displayed no significant differences between the two T1 mappings in all groups (*p* > 0.05). Among groups, no significant difference was shown in the liver T1 estimates in both T1 mappings (*p* > 0.05). The liver volume in Group C was larger than that in Groups A and B in both T1 mappings (*p＜0.05*) (Table 4).

**Table 4:** Variability of the entire liver T1 estimates and volumes between the T1 mappings of FB stack-of-stars FSPGR and BH FSPGR and among three groups (n = 75).

**Group**	**T1 estimates (ms)** **(95%*****CI*****)**				**Volumes (cm**^**3**^**)** **(95%*****CI*****)**			
	**FB FSPGR**	**BH FSPGR**	***t***	***p***	**FB FSPGR**	**BH FSPGR**	***t***	***p***
Group A	880.51 ± 86.56 (822.36–922.67)	854.51 ± 85.71 (796.93–932.09)	0.976	0.352	966.72 ± 240.21 (805.334–1128.09)	965.37 ± 222.81 (815.69–1115.05)	0.047	0.964
Group B	872.36 ± 120.45 (768.96–970.24)	876.50 ± 125.54 (802.57–950.89)	−0.258	0.798	1169.80 ± 376.14 (1026.73–1312.88)	1152.27 ± 412.70 (995.29–1309.25)	0.369	0.715
Group C	846.45 ± 115.03 (796.94–855.97)	821.10 ± 131.17 (776.04–866.15)	1.710	0.096	1466.04 ± 377.05 (1336.52–1595.55)	1468.55 ± 369.02 (1345.22–1591.88)	−0.101	0.920
*Z*	1.352	2.844			**18.592**	**22.421**		
*p*	0.509	0.241			＜0.01*	**＜0.01**^**#**^		

Data are shown as mean ± SD; FB, free-breathing; FSPGR, fast spoiled gradient recalled-echo; BH, breath-hold; *CI*, Confidence Interval.

*, Group A＜Group C (*Z* = −27.751,*p* = 0.001), Group B＜Group C (*Z* = −18.218,*p* = 0.003).

^#^, Group A＜Group C (*Z* = −30.779,*p*＜0.001), Group B＜Group C (*Z* = −19.729,*p* = 0.001).

The ratios of |T1_liver_-T1_sat_|/T1 _liver_ in FB sequence were higher than those of BH FSPGR in the overall enrolled population and in Group C (*p* both < 0.05) (Table 5).

**Table 5:** Variability of the ratio of |T1_liver_-T1_sat_|/T1_liver_ of the entire liver between the T1 mappings of FB stack-of-stars FSPGR and BH FSPGR (n = 75).

**Group**	**|T1**_**liver**_**-T1**_**sat**_**|/T1**_**liver**_ **(95%*****CI*****)** **FB FSPGR**	**|T1**_**liver**_**-T1**_**sat**_**|/T1**_**liver**_ **(95%*****CI*****)** **BH FSPGR**	***t***	***p***
All participants	0.537 ± 0.066 (0.524–0.556)	0.490 ± 0.105 (0.466–0.514)	**3.137**	**0.002**
Group A	0.493 ± 0.075 (0.442–0.543)	0.429 ± 0.155 (0.325–0.533)	1.013	0.105
Group B	0.542 ± 0.065 (0.517–0.566)	0.510 ± 0.086 (0.478–0.543)	1.787	0.085
Group C	0.554 ± 0.068 (0.530–0.577)	0.492 ± 0.096 (0.459–0.525)	**2.415**	**0.026**

Data are shown as mean ± SD; *CI*, Confidence Interval.

For the entire liver avoiding obvious motion artifacts of the 29 participants with BH failure, there was no significant difference in the T1 estimates (884.97 ± 124.99, 95%*CI* [837.42–932.51] vs. 873.11 ± 110.18, 95% *CI* [831.20–915.02], *t* = −0.758, *p* = 0.455) and in the volumes (1217.11 ± 393.22, 95% *CI* [1070.28–1363.94] vs. 1172.33 ± 418.90, 95% *CI* [1015.91–1328.75], *t* = 0.995, *p* = 0.328) between the FB stack-of-stars FSPGR and BH FSPGR.

There was a positive correlation between BMI and the whole liver volume in the FB sequence (*r* = 0.544, *p*＜0.001) and the BH sequence (*r* = 0.622, *p*＜0.001). There was no correlation between BMI and the liver T1 estimates in FB or BH sequence. BMI had a positive weak correlation with the liver PDFF (*r* = 0.268, *p* = 0.02). The higher the liver PDFF, the larger the liver volume in the FB sequence (*r* = 0.598, *p*＜0.001) and the BH sequence (*r* = 0.594; *p*＜0.001). There was a negatively moderate correlation between the liver PDFF and the T1 estimates in both sequences (FB stack-of-stars FSPGR, *r* = −0.742, *p*＜0.001; BH FSPGR, *r* = −0.646, *p*＜0.001).

### 3.3 Qualitative evaluation of the two T1 mappings

The inter-readers consistencies of all visual assessments were good to excellent for both sequences (κ = 0.699–0.831, *p* all< 0.001) (Table 6).

**Table 6:** Inter-readers variability of qualitative image quality grading for FB stack-of-stars FSPGR and BH FSPGR.

**Sequence**	**Quality Parameter**	**Weighted Kappa**	***95% CI***	***p***
FB stack-of-stars FSPGR	Streak artifacts/Motion artifacts	0.739	0.546–0.932	**< 0.001**
	Liver boundaries	0.798	0.764–0.923	**< 0.001**
	Overall image quality	0.919	0.763–1.076	**< 0.001**
BH FSPGR	Streak artifacts/Motion artifacts	0.798	0.644–0.952	**< 0.001**
	Liver boundaries	0.831	0.701–0.962	**< 0.001**
	Overall image quality	0.892	0.771–1.012	**< 0.001**

FB, free-breathing; FSPGR, fast spoiled gradient recalled-echo; BH, breath-hold; CI, Confidence Interval; Two readers’ experience: two radiologists with 6- and 9-years’ abdominal diagnosis experience.

**Fig. 6:**
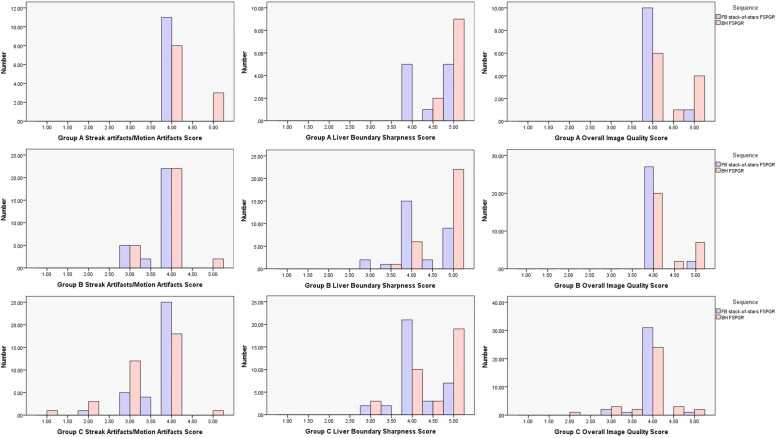
The score distribution of the three groups in T1 map of FB stack-of stars FSPGR and BH FSPGR in terms of streak artifacts/motion artifacts, liver boundary sharpness and overall image quality. FB, free-breathing; FSPGR, fast spoiled gradient recalled-echo; BH, breath-hold.

**Fig. 7:**
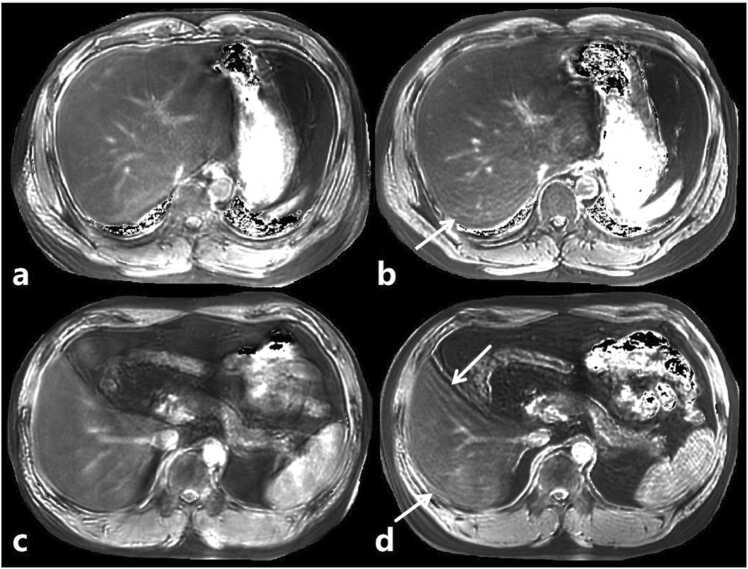
Paired T1 maps acquired from identical anatomic slices in a 33-year-old female patient (BMI =30.35 kg/m²) for direct comparison between two acquisition strategies: free-breathing stack-of-stars FSPGR (a, c) and conventional breath-hold FSPGR (b, d). (a, c) FB-derived T1 maps show homogeneous signal distribution with minimal streak or motion-related artifacts. (b, d) Corresponding BH FSPGR T1 maps present obvious respiratory motion artifacts at peripheral hepatic margins (white arrows), which distort liver edge definition and local signal uniformity. FB, free-breathing; FSPGR, fast spoiled gradient recalled-echo; BH, breath-hold, BMI, body mass index.

The score distribution of the three groups in the two T1 mappings is shown in Fig. 6. Comparing with FB stack-of-stars FSPGR, the BH sequence received significantly higher rating scores for overall image quality in Group B and for liver boundary sharpness in all groups (*p* < 0.05). For Group C, more severe motion artifacts were present in BH FSPGR (*p* = 0.015) (Table 7). Among groups, significant differences were observed with regard to the overall image quality (Group C＜Group B, *Z* = 11.240, *p* = 0.014 and Group C＜Group A, *Z* = −16.439, *p* = 0.009) and artifacts (Group C＜Group B, *Z* = 11.544, *p* = 0.013 and Group C＜Group A, *Z* = −22.649, *p*＜0.001) in BH FSPGR. No significant difference was identified in any aspects of qualitative quality evaluations in FB stack-of-stars FSPGR (overall image quality: *H* = 3.472, *p* = 0.176; liver boundary sharpness: *H* = 3.292, *p* = 0.193; and streak artifacts/motion artifacts: *H* = 3.820, *p* = 0.148). Fig. 7 presents the comparison results of the two T1 mappings’ performances for a 33-year-old female (BMI = 30.35 kg/m²). While the FB stack-of-stars FSPGR sequence under stable respiratory conditions yielded uniform mappings with high consistency (a, c), the BH FSPGR mapping suffered from severe motion artifacts (b, d). These artifacts, most pronounced near the liver margin, are attributed to inconsistent breath-holding during the BH acquisition.

**Table 7:** Variability of the entire livers’ average qualitative image quality grading of the two readers between the T1 mappings from FB stack-of-stars FSPGR and BH FSPGR in three groups.

**Group**	**Qualitative Parameter**	**Quality grading scores**# **FB stack-of-stars FSPGR: BH FSPGR**	***Z***	***p***
Group A (N = 11)	Streak artifacts/Motion artifacts	4 (4, 4): 4 (4, 5)	−1.732	0.083
	Liver boundary sharpness	4.5 (4, 5): 5 (5, 5)	−2.251	**0.024**
	Overall image quality	4 (4, 4): 4 (4, 5)	−1.444	0.149
Group B (N = 29)	Streak artifacts/Motion artifacts	4 (3.75, 4): 4 (4, 4)	1.897	0.058
	Liver boundary sharpness	4 (4, 5): 5 (4.5, 5)	−3.717	**< 0.001**
	Overall image quality	4 (4, 4): 4 (4, 4.75)	−2.181	**0.029**
Group C (N = 35)	Streak artifacts/Motion artifacts	4 (3.5, 4): 4 (3, 4)	−2.430	**0.015**
	Liver boundary sharpness	4 (4, 4.5): 5 (4, 5)	−2.180	**0.029**
	Overall image quality	4 (4, 4): 4 (4, 4)	−0.105	0.917

FB, free-breathing; FSPGR, fast spoiled gradient recalled-echo; BH, breath-hold.

# Data demonstrated as Median (P_25_, P_75_) were used to represent the grading scores.

The lower part of the liver received poorer scores for the artifacts than the upper liver in BH FSPGR (*p* < 0.001) and the FB stack-of-stars FSPGR surpassed BH FSPGR in the lower liver for the artifacts (*p* < 0.001) (Table 8). As shown in Table 9, for BH FSPGR, there were significant differences among the three groups in overall image quality grading and in motion artifacts grading. No significant difference was shown in any qualitative evaluation among groups in FB sequence (*p*＞0.05).

**Table 8:** Variability of qualitative image quality grading between the upper and lower liver and between the T1 mappings of FB stack-of-stars FSPGR and BH FSPGR for 29 participants with BH failure.

**Sequence**	**Overall Image Quality**				**Liver Boundaries**				**Streak/Motion Artifacts**			
	**Upper liver**	**Lower liver**	***Z***	***p***	**Upper liver**	**Lower liver**	***Z***	***p***	**Upper liver**	**Lower liver**	***Z***	***p***
FB stack-of-stars FSPGR	4 (4, 4.75)	4 (4, 4)	−0.656	0.512	4 (4, 4)	4 (4, 4)	−0.539	0.590	4.5 (4, 5)	4 (4, 5)	−1.366	0.172
BH FSPGR	4 (4, 4)	4 (4, 4)	−0.964	0.335	5 (4, 5)	5 (4, 5)	−0.029	0.977	4 (4, 4.75)	3 (3, 4)	**−5.554**	**< 0.001**
*Z*	−0.369	−1.667			**−3.464**	**−3.084**			−1.727	**−4.716**		
*p*	0.712	0.096			**0.001**	**0.002**			0.084	**< 0.001**		

Data shown as Median (P25, P75) were calculated by considering the average values of the two readers using a five-point Likert-type scale. FB, free-breathing; FSPGR, fast spoiled gradient recalled-echo; BH, breath-hold.

**Table 9:** Variability of qualitative image quality grading of 29 participants with BH failure among groups in T1 mappings from FB stack-of-stars FSPGR and BH FSPGR.

**Sequence**	**Streak/Motion Artifacts**					**Liver Boundary sharpness**					**Overall Image Quality**				
	**Group A**	**Group B**	**Group C**	***Z***	***p***	**Group A**	**Group B**	**Group** **C**	***Z***	***p***	**Group A**	**Group B**	**Group C**	***Z***	***p***
FB stack-of-stars FSPGR	4 (4,4)	4 (3.5,4)	4 (4,4)	1.650	0.438	5 (5,5)	4.5 (4,5)	4.5 (4,5)	3.042	0.218	4 (4,4)	4 (4,4)	4 (4,4)	4.197	0.123
BH FSPGR	4.5 (4,5)	3 (3,4)	3 (3,4)	**7.708**	0.021*	5 (5,5)	5 (4,4)	4 (3,4.5)	−2.764	0.251	4 (4,4)	4 (3,4)	5 (5,5)	**11.900**	**0.003**#

FB, free-breathing; FSPGR, fast spoiled gradient recalled-echo; BH, breath-hold.

* Group C＜Group B (*Z* = 2.195, *p* = 0.028), Group C＜Group A (*Z* = −2.156, *p* = 0.031).

# Group C＜Group B (*Z* = 2.039, *p* = 0.036), Group C＜Group A (*Z* = −3.148, *p* = 0.002), Group B ＜Group A (*Z* = −2.041, *p* = 0.041).

## 4 Discussion

This study demonstrated the feasibility of generating T1 mappings using the FB radial stack-of-stars FSPGR sequence across participants with varying BMIs, highlighting its particular value for individuals who cannot reliably hold their breath. Although the BH FSPGR sequence tended to yield higher overall visual qualitative scores in certain subgroups, it remained susceptible to motion artifacts, which noticeably compromised image quality. By contrast, the FB approach exhibited more consistent performance in both qualitative and quantitative analyses among obese subjects.

### 4.1 Qualitative evaluation

As a quantitative technique, T1 mapping remains sensitive to image quality, underscoring the importance of qualitative assessment. In the present study, T1 mapping from BH FSPGR received higher ratings in overall image quality for the overweight group and for liver boundary sharpness across all groups. A major limitation of radial k-space sampling is streak artifacts, which arise from undersampling and respiratory motion. Motion-induced signal loss also leads to an overall blurry appearance of images, and these artifacts collectively result in lower scores for edge sharpness [13], [25]. Some previous studies have reached similar conclusions, noting that FB sequences face challenges in outperforming BH sequences in qualitative image quality for both non-enhanced and contrast-enhanced images [26], [27]. Notably, in the BH FSPGR group, image quality—particularly overall impression and motion artifact scores—declined with increasing BMI. Pronounced motion artifacts were frequently observed in the lower liver regions. In contrast, the FB sequence produced consistently diagnostic T1 mappings regardless of the BMI, with a lighter degree of streak artifacts. This is consistent with the well-known increased difficulty of maintaining a consistent breath-hold in obese subjects. In addition, we hypothesize that in obese participants, the extended scan coverage required a longer acquisition window [28], increasing the likelihood of breath-hold failure and consequent image degradation. Besides, the designed FB sequence utilized a higher NEX to enhance its robustness against respiratory motion, a design strength that contributed to its stable performance in the free-breathing setting. Thus, the FB radial technique minimizes motion sensitivity and allows stable image acquisition during free breathing. This aligns with the previous studies by Azevedo RM and Deng HP, et al. in patients with esophageal cancer and in pediatric cohorts, where FB radial MRI outperformed BH methods in cases of respiratory irregularity, effectively mitigating severe artifacts caused by inconsistent breath-holding [27], [29]. In our cohort, 2 BH failures occurred in participants with normal BMI, often attributable to anxiety, transient inability to follow breath-hold commands, or subclinical respiratory variation. The FB sequence matches BH performance and can replace BH for routine liver T1 mapping across all BMI groups, including normal-BMI patients prone to transient breath-hold failure.

### 4.2 Quantitative evaluation

Liver T1 mapping is increasingly recognized as a valuable non-invasive biomarker across a spectrum of hepatic pathologies [18], [29], [30], [31]. The impact of B1 + inhomogeneity is a spatially varying deviation between the actual flip angle experienced by spins in tissue and the nominal flip angle used in the T1 fitting model [19]. This non-linear error corrupts the quantitative accuracy and confounds spatial comparisons, which is particularly problematic for assessing diffuse liver disease, where subtle T1 changes are clinically significant. To mitigate this fundamental vulnerability, we integrated a B1 + mapping acquisition into our protocol. This B1 correction step is essential to ensure that the estimated T1 values reflect true tissue relaxation properties rather than artifacts of the transmit field, thereby enhancing the reproducibility of our quantitative results.

In the present study, no significant differences were observed in whole-liver T1 estimates or liver volume between the two sequences, either in the general cohort or among the 29 participants with breath-hold failure. These findings indicate good measurement consistency between the free-breathing radial FSPGR and conventional breath-hold FSPGR sequences, and the proposed approach exhibits strong robustness against respiratory motion. Across BMI subgroups, no significant differences were detected in liver PDFF or T1 estimates between sequences. Interestingly, high BMI was not invariably associated with elevated PDFF, suggesting variability in metabolic health among individuals with obesity. Although this study lacked additional clinical biomarkers to further elucidate this observation, our data indicated interrelations among BMI, PDFF, and liver volume in both mapping techniques.

To quantify the clarity of T1 maps, a contrast-to-noise ratio (CNR)-like metric was calculated in the present study. FB stack-of-stars FSPGR demonstrated higher values for the overall enrolled population and obese group, and demonstrated comparable results with BH FSPGR in the normal BMI group and overweight group. This observation might be attributed to the capability of FB radial acquisition sequence insensitive to motion artifacts. Additionally, higher temporal resolution in FB radial acquisition FSPGR images might result in higher ratios. Analogous studies on esophageal cancer, stomach cancer, lung nodules, rectal cancer, and pediatric abdominal MRI have reported similar findings that radial acquisition sequences displayed greater signal-to-noise ratio and/or CNR values than the conventional acquisition sequences [8], [17], [25], [32], [33]. Overall, the proposed T1 mapping method presents favorable technical performance for liver imaging, which showed promising potential as an alternative to conventional sequences for liver T1 measurement, overcoming the limitations related to breath holding.

### 4.3 Comparison with MOLLI Techniques

Analysis from both T1 mapping techniques revealed an inverse relationship between liver T1 estimates and PDFF, which is consistent with the results reported by Higashi M et al. using in-phase-based sequences [18]. This association arises primarily from the methodological characteristics of composite water-fat signals, rather than reflecting inherent biological properties of liver tissue. The short intrinsic T1 relaxation time of fat contributes to combined signal alterations in fat-infiltrated liver, leading to the observed correlation [34], [35]. Notably, studies adopting modified Look-Locker inversion recovery (MOLLI) sequences have documented a positive correlation between PDFF and T1 measurements, a trend also observed by Higashi et al. in opposed-phase acquisitions [18], [35], [36]. Such conflicting trends are rooted in the distinct physical principles of different pulse sequences. MOLLI is based on balanced steady-state free precession (b-SSFP), where fat-related off-resonance effects and water-fat phase interference can artificially lengthen the measured T1 relaxation time [37], [38]. These differences clearly indicate that the correlation pattern between T1 and fat fraction is predominantly determined by imaging acquisition schemes and sequence physics [34].

Apart from water-fat signal interactions, the two techniques also differ substantially in other methodological and practical aspects. Benefiting from the inversion-recovery design, MOLLI is less susceptible to B1 + inhomogeneity. By contrast, VFA methods are intrinsically sensitive to B1 + variation [15]; nevertheless, T1 quantification can be reliable when B1 + correction with validated mapping approaches is applied.

In terms of motion performance, standard single-slice MOLLI enables fast acquisition but may cause sampling bias and cannot fully reflect heterogeneous fat distribution across the liver. Multi-slice MOLLI requires multiple breath-holds, which easily results in slice misregistration and places a heavy burden on patients with obesity, young age, advanced age or respiratory dysfunction. In contrast, volumetric free-breathing stack-of-stars VFA achieves full hepatic coverage and effectively alleviates motion artifacts. However, this technique has inherent limitations, including a trade-off between scan duration and spatial resolution. Its relatively long acquisition time may restrict its routine clinical workflow applicability [17]. Notably, ongoing advances in MRI reconstruction algorithms are expected to reduce sampling views while maintaining diagnostic image quality, which can effectively shorten scan time and further improve the clinical practicability of this technique.

### 4.4 Clinical implications

By validating a free-breathing, VFA T1 mapping technique with integrated B1 + correction—an approach that is both technically robust and clinically practical—this study addresses a critical barrier to the widespread adoption of quantitative liver MRI, particularly for patients unable to sustain breath-holds. While this work focuses on obese patients with potential liver disease, the proposed free-breathing technique offers a practical and robust alternative to conventional breath-hold imaging and is broadly applicable to other patients requiring abdominal MRI who cannot tolerate prolonged breath-holding, such as the elderly, young children, critically ill individuals, those with impaired respiratory function, and patients with malignant tumors. It provides a reproducible free-breathing alternative for hepatic T1 mapping, supporting non-invasive abdominal imaging assessment across diverse patient populations and clinical settings.

### 4.5 Limitations

This study has several limitations. First, all participants were recruited from a single institution and scanned on a unified 3.0 T MRI system, which limits the generalizability of our findings to other scanner platforms, magnetic field strengths, and patient populations from different medical centers. This single-center design was constrained by clinical practicality and ethical approval scope in the current research, and further multi-center enrollment and large-scale subgroup validation will be required to consolidate and extend the conclusions. Second, the overall sample size is limited, especially for the normal-BMI subgroup, rendering the corresponding findings exploratory. Future large-scale prospective studies with expanded BMI-stratified cohorts are required to validate our results, improve statistical robustness, and verify the general applicability of the inter-sequence consistency. Third, this study only performed a head-to-head comparison of two FSPGR sequences and did not incorporate other mainstream T1 mapping techniques, such as MOLLI, for cross-validation and comprehensive performance evaluation. Future studies will include multi-technique comparisons with established T1 mapping sequences and conduct histopathological validation to further validate the clinical practicability and superiority of the proposed FB sequence. Fourth, neither sequence included fat suppression or water-fat separation. In this obese cohort with hepatic fat accumulation, T1 values represent combined water-fat signals, limiting the clinical interpretability of liver T1 estimates for tissue characterization, though the sequence comparison itself remains valid. Future work will explore fat-corrected T1 mapping. Finally, by excluding patients with hepatic comorbidities, our results may not extend to livers with significant pathology.

## 5 Conclusion

In conclusion, T1 mapping via the VFA FB radial stack-of-stars FSPGR sequence is a practical clinical alternative for abdominal imaging in overweight and obese individuals, effectively addressing the key challenge of BH failure.

## Author Agreement

This manuscript is approved by all authors for publication. We would like to declare on behalf of my co-authors that the work described was original research that has not been published previously, and not under consideration for publication elsewhere, in whole or in part. All the authors listed have approved the manuscript that is enclosed.

## CRediT authorship contribution statement

**Haiyun Xu:** Writing – original draft, Visualization, Validation, Supervision, Investigation, Formal analysis, Data curation. **Zhijun Hou:** Validation, Supervision, Resources, Investigation. **Ming Zhang:** Visualization, Investigation, Data curation. **Ran Guo:** Visualization, Investigation, Data curation. **Jialu Zhang:** Writing – review & editing, Visualization, Validation, Software. **Guifeng Fu:** Visualization, Validation, Software, Investigation. **Qingqing Wen:** Visualization, Validation, Software, Investigation. **Zehua Li:** Visualization, Data curation. **Feng Tao:** Validation, Supervision, Resources, Investigation, Formal analysis, Conceptualization, Funding acquisition. **Shuohui Yang:** Writing – review & editing, Supervision, Project administration, Methodology, Funding acquisition, Formal analysis, Conceptualization.

## Ethical approval

This study was approved by the ethics committee of shanghai Municipal Hospital of Traditional Shanghai Municipal Hospital of Traditional Chinese Medicine, [No.2023SHL -KY−84–01], and informed consent was provided by all the patients.

## Funding

This study had received funding by New medical technology research and transformation seed program of Shanghai Municipal Commission of Health and Family (Grant no.: 2024ZZ2047), the Explorers Program of Shanghai (Basic Research Funding) (Grant no.: 25TS1406300), the Three level prevention and treatment of major chronic diseases (coronary heart disease, diabetes, sleep disease) with traditional Chinese medicine intervention and evaluation system of health services (Grant no.: ZY(2025-2027)-1-2-4) and the National Traditional Chinese Medicine Advantage Specialist Construction Project (Grant no.: GKYS4019082026017).

## Declaration of Competing Interest

Consent to participate Informed consent was obtained from all individual participants included in the study.
